# Occult invasive cervical cancer after simple hysterectomy: a multi-center retrospective study of 89 cases

**DOI:** 10.1186/s12885-016-2480-1

**Published:** 2016-07-20

**Authors:** Huimin Bai, Dongyan Cao, Fang Yuan, Huilan Wang, Jie Chen, Yue Wang, Keng Shen, Zhenyu Zhang

**Affiliations:** Department of Obstetrics and Gynecology, Beijing Chao-Yang Hospital, Capital Medical University, Beijing, China; Department of Obstetrics and Gynecology, Peking Union Medical College Hospital, Chinese Academy of Medical Sciences & Peking Union Medical College, Beijing, China; Department of Obstetrics and Gynecology, the Affiliated Hospital of Medical College Qingdao University, Qingdao, China; Department of Obstetrics and Gynecology, the Second Hospital of Hebei Medical University, Shijiazhuang, China; Department of Pathology, Peking Union Medical College Hospital, Chinese Academy of Medical Sciences & Peking Union Medical College, Beijing, China; Department of Pathology, Beijing Chao-Yang Hospital Affiliated China Capital Medical University, Beijing, China

**Keywords:** Cervical cancer, Inadvertent hysterectomy, OICC, Adjuvant treatment, Simple observation

## Abstract

**Background:**

Occult invasive cervical cancer (OICC) is sometimes incidentally found in surgical specimens after a simple hysterectomy (SH). This study was aimed at identifying a subset of patients with OICC who have a favorable prognosis. This patient group may not require adjuvant radiotherapy and other procedures.

**Methods:**

The medical records of women in whom OICC was detected after an inadvertent SH were retrospectively reviewed. The relevant data, including clinicopathological characteristics, treatment and clinical outcome were evaluated. The primary and secondary endpoints were overall survival (OS) and relapse-free survival (RFS), respectively.

**Results:**

Eighty-nine patients who met the inclusion criteria were included for analysis, and the risk of OICC was found to be 1.9 %. Finding an invasive cancer in a hysterectomy specimen after a conization procedure that shows positive margins was the most common reason (41.6 %) for the performance of inadvertent SH. In the univariate analysis, a tumor width > 20 mm, deep stromal invasion, and lymph node metastasis (LNM) were adversely associated with relapse (*P* < 0.001, < 0.001, and = 0.001, respectively) and survival (*P* = 0.003, 0.004, and 0.027, respectively), although these parameters were not independently associated with patient prognoses in the multivariate analysis. In patients with a tumor width ≤ 20 mm and superficial stromal invasion in the observation subgroup, the 5-year RFS and 5-year OS were both 100 %, whereas they were 57.1 % and 66.7 %, respectively, in patients with a tumor size > 20 mm and deep stromal invasion in the radiotherapy or chemotherapy subgroup (*P* < 0.001, and = 0.008, respectively).

**Conclusions:**

Simple observation after a lymphadenectomy procedure may be feasible in OICC patients with a tumor width ≤ 20 mm, superficial stromal invasion, a negative section margin in hysterectomy specimens, and no LNM.

## Background

The optimal treatment for invasive cervical cancer consists of either a radical hysterectomyand pelvic lymphadenectomy or radiotherapy (RT). Comparable rates of survival are obtained when regimens are initiated in early stage disease, regardless of which therapeutic modality is utilized [1]. However, occult invasive cervical cancer (OICC) is sometimes incidentally found in surgical specimens after a simple hysterectomy (SH). SHs are performed for supposedly benign gynecologic conditions and pre-invasive cervical lesions or microinvasive cervical cancer. For lesions that do not qualify as microinvasive, the SH procedure is suboptimal and significantly associated with inferior survival rates [2, 3]. Further treatment, such as RT [4–12] or radical parametrectomy (RP) [13–21], is therefore warranted in these patients. However, a growing number of retrospective studies [22–27], in addition to one of our previously published studies [28], have suggested that RP can be safely omitted in patients with small volume tumors (a largest tumor diameter ≤ 2 cm) and other favorable pathological characteristics, such as superficial stromal invasion (≤10 mm or 5 mm), and negative lymphovascular space involvement (LVSI). All of these selection criteria can be reliably evaluated using SH specimens. Thus, the aim of the present study is to identify a subset of patients with OICC following inadvertent SH who experienced a favorable outcome. This patient group may not require adjuvant RT or other procedures.

## Methods

The medical records of women with an invasive cervical cancer that was detected after inadvertent SH who were treated at one of 4 hospitals from October 2003 to February 2012 were reviewed. The hospitals included The Peking Union Medical College Hospital, Affiliated Hospital of Medical College Qingdao University, The Second Hospital of Hebei Medical University, and Beijing Chao-Yang Hospital, Capital Medical University. Demographic and clinico-pathological data were retrospectively reviewed. Two independent pathologists with extensive backgrounds in gynecologic pathology reviewed all of the pathological slides for the specific purposes of this study and were blinded to patient outcomes. During this review, we collected all missing data on pathologic risk factors.

Tumor width, which was defined as the longest tumor diameter, was evaluated using a postoperative gross measurement before the tissue was fixed. Based on these data, the tumors were divided according to size as either ≤ 2 cm or > 2 cm. Stromal invasion depth was measured perpendicularly from the basement membrane of the surface epithelium using an ocular micrometer. The degree of stromal invasion was classified as either deep or superficial based on its depth (>5 mm or ≤ 5 mm, respectively). If there was substantial residual tumor in an RH specimen after a previously performed conization, the longest diameter was determined to be the sum of the tumor width of the cone biopsy plus that of the RH specimen obtained from the conization site. Similarly, the maximum depth of invasion was determined as the sum of the invasion depth observed in the cone biopsy and that observed in the RH specimen obtained from the conization site. LVSI was defined as the unequivocal presence of malignant cells in endothelial-lined spaces as observed upon a histological examination of the specimen. Marginal status was interpreted as positive when an invasive carcinoma or an in situ carcinoma was found in a parametria or vagina of a hysterectomy specimen. Patients with microinvasive disease (stromal invasion depth ≤ 3 mm) and no LVSI were excluded from the analyses because SH alone is the definitive treatment for this group and is not associated with any safety concerns [29, 30].

Adjuvant treatment after SH included pelvic lymphadenectomy with or without para-aortic lymphadenectomy and RT or concurrent chemoradiotherapy (CCRT). RT was administered in patientswith at least 1 high-risk pathological factor, such as vaginal invasion, parametrial invasion, or lymph node metastasis (LNM), and patients with 2 intermediate-risk factors for relapse, including LVSI, deep stromal invasion or microscopic metastasis in > 2 lymph nodes [31, 32]. The RT treatment consisted of whole pelvic RT with a total dose of 45 Gy that was divided into 20–30 applications. For CCRT patients, cisplatin (40 mg/m^2^) was administered weekly. For patients who failed to meet the above criteria, the decision to administer RT was generally based on the patient’s age, their informed consent, and the doctor’s recommendation.

After the completion of treatment, the women were followed up every 3 months during the first year, every 4 months during the second year and every 6 months thereafter. A ThinPrep cytologic test was performed every 6 months. In women for whom regular follow-up information was not available, an effort was made to contact these patients by telephone or correspondence to obtain this information. Relapse was defined by clinical or imaging evidence and was confirmed pathologically. Local recurrence (LR) was defined as recurrence in the pelvis, and relapse-free survival (RFS) was defined as a lack of LR and distant metastasis. RFS times were calculated as the period between the date of the initial surgery and the date of relapse. Women who were disease-free at the time of their last visit were censored. Overall survival (OS) times were calculated in months from the date of the initial surgery to the date of patient death from the disease. Patients who died of other conditions or had survived at the time of their last visit were censored.

All statistical analyses were performed using SAS® Version 9.2 (SAS Institute, Cary, NC). All tests were 2-sided, and *P*-values ≤ 0.05 were considered statistically significant. The frequency distributions of clinicopathological parameters were compared between groups using Chi-square or Fisher’s exact tests, and a Kruskal-Wallis test was used to compare mean values. The Kaplan-Meier method was used to analyze relapse and survival times. A log rank test was used to compare different survival curves. A Cox proportional hazards model was applied to all of the parameters that were found to be significant in the univariate analysis.

## Results

During the study period, 4635 consecutive patients with cervical cancer were treated at the four hospitals. One hundred and twenty-eight women were diagnosed with cervical cancer after inadvertent SH. Thirty-one of these patients had microinvasive disease (stromal invasion depth ≤ 3 mm) but were negative for LVSI and were therefore excluded. Eight patients without complete medical records or follow-up information were also excluded. Consequently, 89 (1.9 %) women were eligible for this analysis. Twenty-four (27.0 %) patients were referred to the four hospitals after their initial hysterectomy.

All hysterectomies were performed by gynecologists or gynecological oncologists. The reasons and indications for performing an inadvertent SH are shown in Table 1. Fourteen patients (15.7 %) had no preoperative Pap smear. Eleven (12.4 %) women had a normal cytology, but the Pap smears were incompletely evaluated in 3 of these patients. An abnormal cytology (≥ ASCUS or AGC) was identified in 56 patients who underwent a colposcopy-directed biopsy. Cervicitis or CIN1-2 was diagnosed in 14 cases (15.7 %), and pre-invasive or microinvasive cervical cancer was diagnosed in 42 cases using this procedure. An indicated conization procedure was not performed in 5 (5.6 %) cases. Thirty-seven (41.6 %) women were diagnosed with a pre-invasive or microinvasive cervical cancer following a conization. These patients showed positive margins in the conization specimen and residual invasive cancer was observed in their hysterectomy specimens. This was the most common reason for performing inadvertent SH.

**Table 1:** Reasons, indications and surgical approaches used for inadvertent hysterectomy in patients who underwent surgery because of a cervical cancer finding

Parameters		No.	%
Reasons			
No preoperative Pap smear		14	15.7
	Negative fractional curettage	4	4.5
	Emergent operation	3	3.4
	Absence of preoperative cytology and histology of the cervix	7	7.9
Negative Pap smear		11	12.4
	False negative	8	9.0
	Incompletely evaluated	3	3.4
Colposcopy-directed biopsy		14	15.7
	Cervicitis	6	6.7
	CIN1-2	8	9.0
Conization procedure		42	47.2
	Pre-insive or microinvasive cervical cancer	37	41.6
	Failure to perform an indicted conization	5	5.6
Data not available		8	9.0
Indications			
Pre- or microinvasive cervical cancer		42	47.2
	Pre-insive	26	29.2
	Microinvasive	16	18.0
Benign disease		47	52.8
	Leiomyoma	16	18.0
	Adnexal mass	12	13.5
	Adenomyosis	11	12.4
	Abnormal uterine bleeding	6	6.7
	Prolapse	5	5.6
Surgical approach			
Laparotomy		43	48.3
Laparoscopy		31	34.8
Transvagina		15	16.9

Pre-invasive or microinvasive cervical cancer was the most common indication (47.2 %) for hysterectomies. The remaining 47 patients were preoperatively diagnosed as having benign disease. All of the hysterectomies were total, including intrafascial hysterectomies which were performed in 16 cases (18.0 %). Thirty-six patients (40.4 %) underwent unilateral (2 cases) or bilateral (34 cases) salpingo-oophorectomy during hysterectomy. None of the 89 patients had macroscopic disease after SH, as determined during a review of the records for initial hysterectomies, clinical examinations, and imaging scans, such as ultrasonic inspections, CT or MRI, and sometimes PET-CT.

The demographic and clinicopathological characteristics of these patients are summarized in Table 2. The mean age at diagnosis was 45.2 years old. The most common histology of the tumors (84.3 %) was squamous cell carcinoma (SCC). The average tumor width was 12.5 mm, and the average stromal invasion depth was 5.6 mm.

**Table 2:** Clinicopathological characteristics of the the included 89 patients

Parameter		Number of patient	Percent (%)
Age (Mean, range)		45.2 ± 11.9, (29–72)	
	≤45	52	58.4
	>45	37	41.6
Histology			
	SCC^a^	75	84.3 %
	AD^b^	12	13.5 %
	ASC^c^	2	2.2 %
Grade			
	1	30	33.7
	2	46	51.7
	3	13	14.6
Tumor width (Mean, range)		12.5 ± 9.1, (3–35)	
	≤20 mm	69	77.5
	>20 mm	20	22.5
Stromal invasion depth (Mean, range)		5.6 ± 3.9, (2–21)	
	≤5 mm	66	74.2
	>5 mm	23	25.8
LVSI^d^			
	+	22	24.7
	-	67	75.3
Positive margin in hysterectomy specimens		3	3.4
	Vaginal invasion	1	1.1
	Parametrial invasion	2	2.2
Lymphadenectomy			
	+	76	85.4
	-	13	14.6
LNM^e^		6	7.9
	Pelvic	6	7.9
	Para-aortic	1	1.3
Adjuvant therapy			
	RT^f^	36	40.4
	CT^g^	26	29.2
	Observation	48	53.9
Follow-up (Month, range)		52.7 ± 27.2, (7–120)	
Relapse		4	4.5
Status at last contact			
	NED^i^	85	95.5
	AWD^j^	1	1.1
	DOD^k^	3	3.4
5-year RFS^l^ (%)		93.9	
5- year OS^m^ (%)		94.7	

Note: ^a^Squamous cell carcinoma; ^b^Adenocarcinoma; ^c^Adenosquamous carcinoma; ^d^Lymph node metastasis; ^e^Lymphovascular space involvement; ^f^Radiotherapy; ^g^Chemoradiation therapy; ^h^Chemotherapy; ^i^No evidence of the disease; ^j^Alive with the disease; ^k^Dead of the disease; ^l^Relapse -free survival; and ^m^Overall survival

Pelvic lymphadenectomy was performed in 76 cases (85.4 %) an average of 23 days after the initial surgery (range: 14–47 days), and 15 of these patients underwent simultaneous para-aortic lymphadenectomy. Lymphadenectomy was omitted in 12 women who underwent RT or CCRT directly. One patient refused to receive further lymphadenectomy and indicated RT, and this patient eventually died of the disease. Six patients underwent bilateral ovarian transposition during lymphadenectomy. The mean number of lymph nodes removed was 23.2 ± 6.0 per patient (range: 7–44). LNM was found in 6 (7.9 %) women, resulting in a total of 32 positive nodes. The mean delay time from hysterectomy to RT, which was performed in 36 (40.4 %) patients, was 47 (range 15–64) days. Scheduled RT was delayed in 2 (7.7 %) patients because of severe radiation enteritis. CT was performed in 26 (29.2 %) cases. Forty-eight (53.9 %) patients did not receive any adjuvant treatment.

The mean follow-up period was 52.7 months. Nine (25 %) patients suffered late complications related to RT that required further management. These complications included frozen pelvis (4 cases), radiation cystitis (3 cases), intestinal obstruction (3 cases), radiation proctitis (2 cases), and rectovaginal fistula (1 case). Two patients with intestinal obstruction required surgical management, and one of them developed short bowel syndrome postoperatively. During the follow-up period, 4 (4.5 %) patients experienced a relapse. The disease recurred locally in 1 case, at distant sites in 2 cases, and both locally and at distant sites in 1 case (Table 3). The distant metastasis sites included the lungs (2 cases), sternum (1 case) and left supraclavicular lymph nodes (1 case). The mean relapse interval was 24.0 months. Three (3.4 %) women died of multiple metastases of the disease, and one (1.1 %) patient was living with the disease at last contact. In total, 85 (95.5 %) women were alive without any evidence of residual tumor at the time of their last visit (Table 2). The 5-year RFS and 5-year OS rates were 93.9 and 94.7 %, respectively, for the entire series. A tumor width > 20 mm, deep stromal invasion, and LNM were identified as significant risk factors for relapse (*P* < 0.001, < 0.001, and = 0.001, respectively) and survival (*P* = 0.003, 0.004, and 0.027, respectively; Table 4) in the univariate analysis. No parameter was identified as an independent risk factor for relapse or survival in the multivariate analysis. All of the patients with a tumor width ≤ 20 mm and superficial stromal invasion had a clear section margin in their hysterectomy specimens and had no positive lymph node when lymphadenectomy was performed. None of the patients in this group developed recurrence or died of the disease (0/56).

**Table 3:** The characteristics of patients who developed recurrence after adjuvant therapy

Patient	Age (years)	Histology	Grade	Tumor width (mm)	Stromal invasion depth (mm)	LVSI	SM	LNM	Adjuvant treatment	Recurrence sites	RFS (months)	OS (months)	Status
1	≤45	SCC	2	33	13	+	-	+	CCRT	Local	35	35	AWD
2	>45	AD	2	33	21	+	-	+	CCRT	Distant	18	43	DOD
3^*^	>45	SCC	1	34	9	-	+	unknown	Obs	Both	9	25	DOD
4	≤45	SCC	2	28	6	-	-	unknown	CCRT	Both	34	54	DOD

Note: *This patient refused lymphadenectomy and RT or CT

**Table 4:** Risk factors related to relapse and survival

Parameter		Relapse		*P* value^a^	DOD		*P* value^a^
		+	-		+	-	
Age							
	≤45	2	50	0.720	2	51	0.417
	>45	2	35		1	35	
Tumor width							
	≤20 mm	0	69	<0.001	0	69	0.003
	>20 mm	4	16		3	17	
Histology							
	SCC	3	72	0.586	2	73	0.421
	AD and ASC	1	13		1	13	
Grade							
	1	1	29	0.725	1	29	0.901
	2 + 3	3	56		2	57	
Stromal invasion depth							
	≤5 mm	0	66	<0.001	0	66	0.004
	>5 mm	4	19		3	20	
LVSI							
	+	2	20	0.335	1	21	0.851
	-	2	65		2	65	
Section margin							
	+	1	2	0.095	1	2	0.079
	-	3	83		2	84	
LNM							
	+	2	4	0.001	1	5	0.027
	-	0	70		0	70	
Adjuvant therapy							
	Observation	1	47	Reference	1	47	Reference
	RT	3	33	0.135	2	34	0.420
	CT	3	23	0.086	2	24	0.294

Note: ^a^Log rank test

**Fig. 1:**
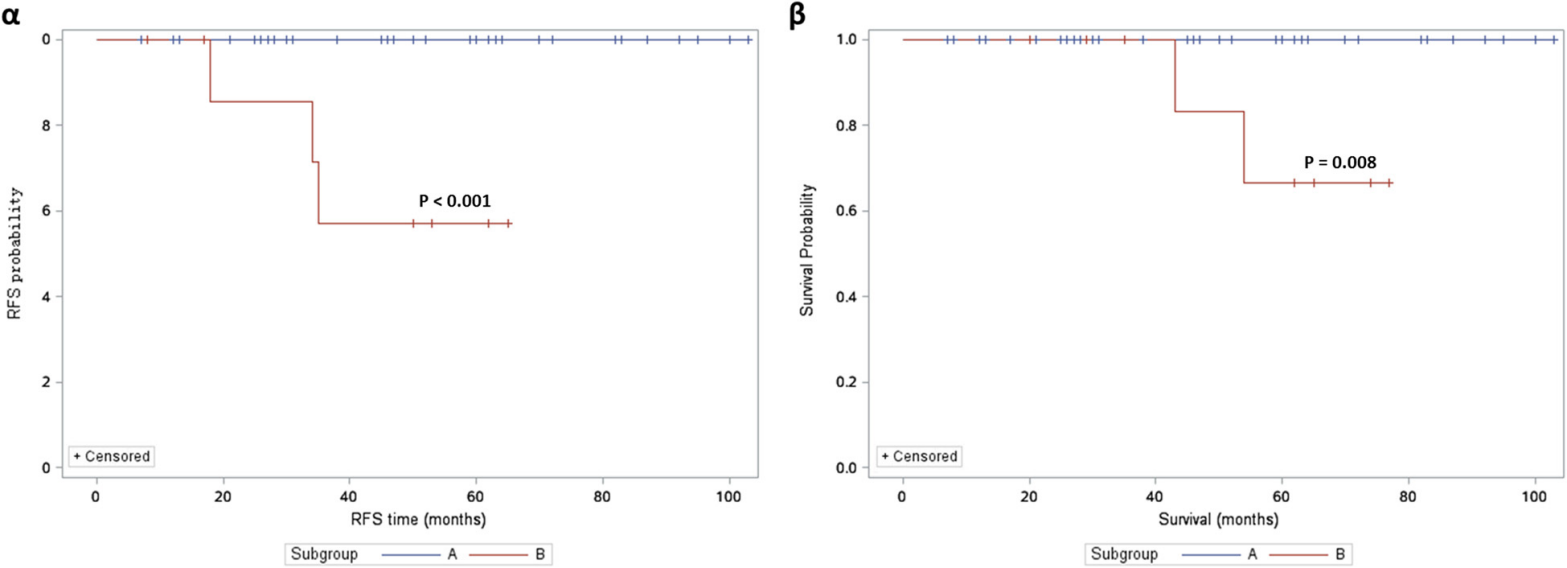
A comparison of relapse (Fig. 1-*α*) and survival curves (Fig. 1-*β*) between Subgroup A and Subgroup B. None of the patients had developed relapse or died of the disease at the last contact in patients in Subgroup A. In contrast, the 5-year RFS and 5-year OS in the patients in Subgroup B were 57.1 % and 66.7 %, respectively (*P* < 0.001, *P* = 0.008, respectively). Note: Subgroup A: patients with a tumor width ≤ 20 mm and superficial stromal invasion in the observation subgroup (*n* = 44). Subgroup B: patients with a tumor size > 20 mm and deep stromal invasion in the RT or CT subgroup (*n* = 9)

Patients in the observation (Obs) subgroup had a slightly more favorable prognosis than those who received RT or CT (5-year PFS: 97.8 % vs 87.7 % and 83.8 %, respectively, *P* = 0.172; 5-year OS: 97.4 % vs 90.3 % and 87.9 %, respectively, *P* = 0.545). The clinicopathological characteristics of the Obs, RT and CT subgroups were compared and are shown in Table 5. Patients with factors associated with a more favorable prognosis, such as a tumor width ≤ 20 mm, superficial stromal invasion, and negative LNM (*P* = 0.001, < 0.001, and = 0.004, respectively) were more likely to be managed using observation. In addition, RT with or without CT was more likely to be performed in elderly women (*P* = 0.002), possibly because of the adverse effect of RT on ovarian function. In the observation subgroup, no patient (0/44) developed relapse or died of the disease among the patients with a tumor width ≤ 20 mm and superficial stromal invasion. In contrast, in RT or CT subgroup, 3 of 9 patients with a tumor size > 20 mm and deep stromal invasion developed relapse, and 2 of them died of the disease. The 5-year RFS and 5-year OS in this latter patient group were only 57.1 and 66.7 %, respectively (*P* < 0.001 and = 0.008, respectively; Fig. 1-αandβ). Thus, RT or CT can be safely omitted in patients with a tumor width ≤ 20 mm, superficial stromal invasion, and negative LNM.

**Table 5:** Comparison of clinic0-pathological characteristics of patients in the RT, CT and Obs subgroups

Parameter		Obs	RT	CT	*P* value
Age					
	≤45	34	13	10	0.002^a^
	>45	14	23	16	
Histology					
	SCC	42	29	22	0.384^a^
	AD + ASC	6	7	4	
Grade					
	1	21	9	5	0.076^a^
	2 + 3	27	27	21	
Tumor width					
	≤20 mm	44	20	18	0.001^a^
	>20 mm	4	16	8	
Stromal invasion depth					
	≤5 mm	47	15	14	<0.001^a^
	>5 mm	1	21	12	
LVSI					
	+	9	12	6	0.301^a^
	-	39	24	20	
Positive margin in hysterectomy specimens					
	+	1	2	2	0.396^a^
	-	47	34	24	
LNM					
	+	0	6	6	0.004^a^
	-	47	18	15	
Follow-up duration (Mean, range); months		52.9 ± 29.4, (7–103)	53.0 ± 23.5, (20–120)	50.9 ± 26.0, (17–105)	0.939^b^
Recurrence, *n* (%)		1 (2.1)	3 (8.3)	3 (15.4)	0.208^a^
Death, *n* (%)		1 (2.1)	2 (5.6)	2 (11.5)	0.495^a^
5-year RFS (%)		97.8	87.7	83.8	0.172^c^
5-year OS (%)		97.4	90.3	87.9	0.545^c^

Note: ^a^Chi-square test or Fisher’s exact test; ^b^Kruskal-Wallis Test; ^c^Log rank test

## Discussion

OICC is not rare, and there is no well-established optimal approach for treating this disease. The best way to manage this clinical dilemma is to prevent its occurrence. OICC has mainly been attributed to inadequate preoperative workup. Its reported incidence was as high as 12.9 % several decades ago [33, 34], but it has significantly dropped to 2.7 % from 6.4 % in more recent studies [14, 16, 35] and was 1.9 % in the present series. This improvement is possibly because of the development of improved screening technologies that can better detect cervical cancer, and an enhanced awareness in both patients and doctors about disease prevention. The vast majority of cases are now diagnosed preoperatively. Based on our data, no preoperative cytology, incomplete preoperative evaluation, and a false negative Pap smear accounted for 28.1 % of the reasons for performing an inadvertent hysterectomy. Finding an invasive cancer in a hysterectomy specimen after a conization procedure with positive margins was the most common reason (41.6 %) for performing an inadvertent SH. A positive margin in a conization specimen suggests the presence of residual disease in a hysterectomy specimen [36]. However, conization lacks sufficient negative predictive value to detect pathological parameters in hysterectomy specimens under these conditions [37, 38]. Thus, when patients are diagnosed with pre-invasive or microinvasive carcinoma of the cervix after a conization and show positive margins in the conization specimen, secondary conization or radical hysterectomy was recommended.

The current treatments available for OICC after SH include RP and partial vaginectomy with pelvic lymphadenectomy, or adjuvant RT with or without CT. None of the patients underwent RP in our series, and we therefore have no direct experience with this management strategy. The reported operative morbidities in RP range widely, from 8.7 to 30 % [16]. The relatively high morbidity rates were mainly attributed to the difficulty of administering RP after SH because of the paucity of tissue that can be removed during the operation [15]. The success of this procedure largely depends on the surgeon’s experience and skill [8, 35]. The rate of serious late complications associated with RT or CCRT in our series was as high as 27 %, which was in agreement with the results described in Park’s report [16].

Women in whom small volume (≤2 cm) tumors were observed who also simultaneously presented with other favorable factors were defined as low risk for parametrial invasion and LNM and were considered to have a favorable prognosis [22–27]. Most of the patients with OICC had small volume tumors, which might also have potentially contributed to their misdiagnosis before SH. The mean tumor width was 12.5 mm, and 77.5 % of the patients had a tumor width ≤ 2 cm in our series. The rates of vaginal invasion and parametrial invasion were only 1.1 and 2.2 %, respectively. However, these data might be underestimated to some extent, because RP and partial vaginectomy were not performed in our series. Several previous studies had similar results [16, 35, 39]. Thus, this patient group might benefit from conservative treatment, such as simple observation.

The reported 5-year OS in OICC patients (after RT or CCRT) in the literature ranges from 54 to 94 % [5–10, 16, 17, 40]. In the present work, the 5-year RFS and 5-year OS were 87.7 and 90.3 %, respectively, in the RT subgroup and 83.8 % and 87.9 %, respectively, in the CT subgroup. A tumor width > 20 mm, deep stromal invasion, and LNM were identified as significant risk factors for relapse and survival in the univariate analysis. The roles that these risk factors play in predicting prognoses in patients with early-stage cervical cancer have been demonstrated in an accumulating number of retrospective studies that have been published in the literature [22–28]. These data were not validated in this study using a multivariate analysis, because of limitations that were associated with the relatively small sample size of our series. According to our data, patients with a tumor width ≤ 20 mm and superficial stromal invasion all had hysterectomy specimens that showed a clear section margin in their hysterectomy specimens, and had no LNM when lymphadenectomy was performed. Among the patients who had these favorable prognostic factors in the observation subgroup, none developed recurrence or died of the disease at the last contact in patients who had these favorable prognostic factors in the observation subgroup. In contrast, the 5-year RFS and 5-year OS were as low as 57.1 and 66.7 % in patients with a tumor size > 20 mm and deep stromal invasion in either the RT or the CT subgroups. These results suggest that it may be safe to omit RT or CT in patients with a tumor width ≤ 20 mm, superficial stromal invasion, a negative section margin, and negative LNM.

LNM was also uncommon in OICC and was found in only 6.7 % of cases in the present study. Omitting lymphadenectomy in early-stage cervical cancer was another attractive option. Kodama et al. [41] reviewed the literature and demonstrated that the rate of LNM was 2.0 % in patients with a tumor size < 2 cm and negative LVSI, and this finding was also confirmed in our previous study [28]. Based on the present data, none of the patients with a tumor width ≤ 20 mm and superficial stromal invasion had a positive lymph node when a lymphadenectomy was performed. However, nodal status has been directly correlated with patient survival [28, 42]. Performing MRI examinations and/or biopsies to evaluate sentinel lymph nodes both offer the potential of improving the sensitivity of pelvic lymph node evaluations [43]. Omitting lymphadenectomy procedures could be cautiously applied in patients with low-risk factors for LNM who give fully informed consent. Otherwise, this procedure should continue to be conserved, either to provide prognostic information or to guide post-operative treatment.

Despite the limitations of its retrospective nature, the present study was conducted using a multi-center design and relatively complete clinicopathological reports and follow-up information. In addition, the data in this analysis spans the most recent ten years, and therefore reflect the latest treatment outcomes for this disease.

## Conclusions

Adjuvant RT or CT could be safely omitted in patients with OICC who also have a tumor width ≤ 20 mm, superficial stromal invasion, no LNM and a negative section margin in a hysterectomy specimen. However, further studies with a larger sample population are warranted to validate these results.

## Abbreviations

CCRT, concurrent chemoradiotherapy; CT, chemotherapy; LNM, lymph node metastasis; LR, local recurrence; LVSI, lymphovascular space involvement; Obs, observation; OICC, occult invasive cervical cancer; OS, overall survival; RFS, relapse-free survival; RP, radical parametrectomy; RT, radiotherapy; SCC, squamous cell carcinoma; SH, simple hysterectomy
